# A genome‐wide siRNA screen for regulators of tumor suppressor p53 activity in human non‐small cell lung cancer cells identifies components of the RNA splicing machinery as targets for anticancer treatment

**DOI:** 10.1002/1878-0261.12052

**Published:** 2017-04-11

**Authors:** Ellen Siebring‐van Olst, Maxime Blijlevens, Renee X. de Menezes, Ida H. van der Meulen‐Muileman, Egbert F. Smit, Victor W. van Beusechem

**Affiliations:** ^1^ Department of Pulmonary Diseases VU University Medical Center Amsterdam The Netherlands; ^2^ Department of Medical Oncology VU University Medical Center Amsterdam The Netherlands; ^3^ Department of Epidemiology and Biostatistics VU University Medical Center Amsterdam The Netherlands; ^4^ Department of Thoracic Oncology Netherlands Cancer Institute Amsterdam The Netherlands

**Keywords:** NSCLC, p53 pathway inhibitor, RNA splice factor, RNAi screen, spliceosome

## Abstract

Reinstating wild‐type tumor suppressor p53 activity could be a valuable option for the treatment of cancer. To contribute to development of new treatment options for non‐small cell lung cancer (NSCLC), we performed genome‐wide siRNA screens for determinants of p53 activity in NSCLC cells. We identified many genes not previously known to be involved in regulating p53 activity. Silencing p53 pathway inhibitor genes was associated with loss of cell viability. The largest functional gene cluster influencing p53 activity was mRNA splicing. Prominent p53 activation was observed upon silencing of specific spliceosome components, rather than by general inhibition of the spliceosome. Ten genes were validated as inhibitors of p53 activity in multiple NSCLC cell lines: genes encoding the Ras pathway activator SOS1, the zinc finger protein TSHZ3, the mitochondrial membrane protein COX16, and the spliceosome components SNRPD3, SF3A3, SF3B1, SF3B6, XAB2, CWC22, and HNRNPL. Silencing these genes generally increased p53 levels, with distinct effects on CDKN1A expression, induction of cell cycle arrest and cell death. Silencing spliceosome components was associated with alternative splicing of *MDM4* mRNA, which could contribute to activation of p53. In addition, silencing splice factors was particularly effective in killing NSCLC cells, albeit in a p53‐independent manner. Interestingly, silencing *SNRPD3* and *SF3A3* exerted much stronger cytotoxicity to NSCLC cells than to lung fibroblasts, suggesting that these genes could represent useful therapeutic targets.

**Abbreviations**
hnRNP: heterogeneous nuclear ribonucleoprotein
NSCLC: non‐small cell lung cancer
pre‐mRNA: precursor mRNA
RP: ribosomal protein
SAP: spliceosome‐associated protein
SF: splice factor
snRNA: small nuclear RNA
snRNP: small nuclear ribonucleoprotein
SR: serine/arginine‐rich

## 1 Introduction

Inactivation of the p53 tumor suppressor pathway is common in cancer, including non‐small cell lung cancer (NSCLC), and correlates with poor survival and resistance to therapy (Petitjean *et al*., 2007; Robles and Harris, 2010). Restoration of wild‐type p53 functions is considered a therapeutic option for cancer, based on a number of observations. First, experimental restoration of wild‐type p53 functions induces significant antitumor responses in mouse models, with a variety of underlying mechanisms of tumor regression in different tumor types (Feldser *et al*., 2010; Hill *et al*., 2015; Junttila *et al*., 2010; Martins *et al*., 2006; Ventura *et al*., 2007; Xue *et al*., 2007). The antitumor efficacy of reactivating p53 appears, however, to depend on the genetic context of the cancer cells. In two NSCLC models, only more advanced tumors with increased oncogenic signaling regressed upon wild‐type p53 restoration (Feldser *et al*., 2010; Junttila *et al*., 2010). This might imply that low‐grade cancer cells with insufficient stress signals to activate restored wild‐type p53 protein could escape treatment effect. Second, cancer cells with functional p53 generally appear to be more vulnerable to treatment with chemotherapeutic drugs. Drug activity screens with potential anticancer compounds on the NCI‐60 cancer cell line panel revealed that many anticancer agents in clinical use, in particular DNA‐damaging agents, were more cytotoxic toward cells with an intact p53 pathway (O'Connor *et al*., 1997; Weinstein *et al*., 1997). Although results from preclinical and clinical studies are often contrasting and may depend on the type of induced cell death, the majority of evidence supports the notion that loss of functional p53 associates with resistance to treatment with several cytotoxic drugs (Pirollo *et al*., 2000; Viktorsson *et al*., 2005). Together, this has fueled the idea that reinstating p53 functions is a valuable therapeutic option.

New drugs that activate p53 in cancer cells are being developed with some already in clinical trials (Hong *et al*., 2014; Khoo *et al*., 2014). The main approach to treat cancers that retain wild‐type p53 is to inhibit the function of negative regulators of p53. In this respect, the vast majority of current activities are focused on inhibiting the interaction between p53 and MDM2. Notably, effective elimination of established tumors by activating p53 probably requires an apoptotic or senescence response, rather than induction of cell cycle arrest. Elevating wild‐type p53 levels by inhibiting the p53–MDM2 interaction was shown to consistently induce cell cycle arrest in a panel of cancer cell lines. In contrast, the induction of apoptosis was highly variable, with NSCLC cells showing hardly any apoptotic response (Tovar *et al*., 2006). The biological consequences of wild‐type p53 restoration depend for a large part on the ability of p53 to activate specific target genes. Many different cofactors influence p53 transcriptional activity at different target gene promoters, thereby directing specific cellular responses (Vousden and Prives, 2009). Here, to contribute to development of new treatment options for NSCLC, we set out to identify molecular targets for wild‐type p53 reactivation in NSCLC cells using genome‐wide RNA interference loss‐of‐function screening. This led to the identification of spliceosome genes as putative targets for anticancer treatment. As the selective cytotoxicity of silencing these genes appeared p53 independent, their identification through screening for induction of p53 activity was serendipitous.

## 2 Materials and methods

### 2.1 Cell lines and culture conditions

A549 NSCLC cells and IMR‐90 lung fibroblasts were purchased from the ATCC; NCI‐H292 and NCI‐H1299 NSCLC cells were obtained from G. Peters (Department of Medical Oncology, VUmc). The identity of the NSCLC cell lines was confirmed by STR analysis, performed under contract at BaseClear (Leiden, the Netherlands). Construction of the p53 activity reporter cell line A549/PG13Luc was described previously (Siebring‐van Olst *et al*., 2013); A549/PG13Luc2 and H292/PG13Luc cells were generated using the same procedures. To generate A549/PG13Luc/shp53 cells, A549/PG13Luc cells were transfected with a pLKO.1 vector expressing a short hairpin targeting *TP53* (Open Biosystems, part of GE Healthcare Dharmacon, Lafayette, CO, USA; TRCN0000003756) and a single cell clone was obtained under puromycin selection.

A549 (reporter) cells were cultured in DMEM, NCI‐H1299 and NCI‐H292 (reporter) cells in RPMI1640, and IMR‐90 cells in EMEM. All media were supplemented with 10% fetal bovine serum (Hyclone, GE Healthcare, Logan, UT, USA) and 1% penicillin/streptomycin. Antibiotics were omitted during validation and characterization experiments. All cultures were performed at 37 °C, at 5% CO_2_ in a humidified atmosphere.

### 2.2 siRNA transfection procedures

Forward siRNA transfections were performed one day after seeding cells in 96‐well culture plates (Greiner Bio‐One, Alphen a/d Rijn, the Netherlands; #655180) for cell viability experiments; 96‐well white‐walled culture plates (Greiner Bio‐One, #655095) for luciferase activity assays; or 10‐cm culture dishes (Greiner Bio‐One, #664160) for RNA isolation. siRNA duplexes from the Dharmacon (Lafayette, CO, USA) si*ARRAY* Whole Human Genome siRNA library, individual siGENOME controls targeting *TP53* (M‐003329‐03), *SYVN1* (M‐007090‐01), *PLK1* (M‐003290‐01), nontargeting siRNA controls NT#1 (D‐001210‐01) or NTp2 (D‐001206‐14), or individual siGENOME siRNAs listed in Table S1 (all from Dharmacon) were diluted in siRNA buffer (Dharmacon B‐002000‐UB) and mixed 1 : 1 with transfection reagent diluted in serum‐free culture medium at least 20 min before addition to the cells. siRNA transfection conditions were optimized for each cell line and were as follows. A549 (reporter) cells were seeded at 750 or 1000 cells/well or 600 000 cells/dish and transfected with 25 nm of siRNA and 0.04 or 0.05% Dharmafect 1 (DF1, #T‐2001); NCI‐H292 (reporter) cells were seeded at 1000 cells per well and transfected with 30 nm of siRNA and 0.05% DF1; NCI‐H1299 cells were seeded at 1000 cells per well and transfected with 25 nm of siRNA and 0.06% DF1; and IMR‐90 cells were seeded at 5000 cells per well and transfected with 50 nm of siRNA and 0.15% Turbofect (Thermo Fisher Scientific, Landsmeer, the Netherlands; R0531).

### 2.3 High‐throughput screening procedures

Three individual genome‐wide siRNA discovery screens were conducted on A549/PG13Luc cells using the Dharmacon si*ARRAY* Whole Human Genome siRNA library comprising single‐target pools of four distinct siRNAs targeting 19 574 annotated genes (NCBI RefSeq58). The screening method was described in detail previously (Siebring‐van Olst *et al*., 2013).

A cell viability screen was performed with siRNA reagents selected from the whole genome library. Cells were transfected in a final volume of 100 μL. On the fifth day after transfection, 25 μL of CellTiter‐Blue reagent (Promega, Leiden, the Netherlands) was added and cells were incubated for 4 h. The reaction was terminated by the addition of 60 μL of 3% SDS, and cell viability was determined by measuring fluorescence at 540‐nm excitation and 590‐nm emission wavelengths using a Tecan Infinite F200 reader.

### 2.4 p53 reporter and cell viability assays

Luciferase expression in stable PG13Luc reporter cell lines was measured three days after siRNA transfection or at 0, 24, and 48 h after treatment with 10 μm of pladienolide B (Cayman Chemical, Uden, the Netherlands; No. 16538) as described (Siebring‐van Olst *et al*., 2013). For the transient Cignal p53 Reporter Assay (Qiagen, Venlo, the Netherlands; CCS‐004L), reporter plasmids were transfected two days after siRNA transfection and luminescence was measured using the Dual‐Luciferase Reporter Assay (Promega, E1910) the next day. Cell viability was determined five (IMR‐90) or six (A549 and NCI‐H1299) days after siRNA transfection by adding 20 μL of CellTiter‐Blue reagent and measuring fluorescence after 3 h.

### 2.5 Gene expression analysis by quantitative RT‐PCR

Cells were harvested three days after siRNA transfection, and mRNA was isolated using the RNeasy plus micro kit (Qiagen, #74034). cDNA was prepared with random primers, Superscript III, dNTPs, and RNase out (all from Invitrogen, part of Thermo Fisher Scientific). Real‐time PCR was carried out on a Roche LightCycler 480 using the Qiagen SYBRgreen PCR kit (#204143). For amplification of *IL17A*,* ZNF226*,* HNRNPL*,* XAB2*, and control household gene *GAPDH*, Quantitect primer sets (Qiagen) were used. All other primers were designed using Primer‐BLAST 3.0 and purchased as custom oligonucleotides from Invitrogen. Primer sets were selected on the basis of melting point analysis and correct amplicon length as determined by gel electrophoresis. Primer details are given in Table S1. Knockdown was quantified relative to *GAPDH* using the ΔΔCt method.

### 2.6 Western blot analysis

A549/PG13Luc cells were seeded at 4450 cells per well in 24‐well culture plates (Greiner Bio‐One) and transfected with 25 nm of siRNA and 0.04% DF1. Three days after transfection, cells of four wells were harvested and pooled. Protein was isolated in RIPA buffer, separated on a 10% polyacrylamide gel, blotted onto Immobilon‐P membrane (Millipore, Amsterdam, the Netherlands; IPVH00010), and incubated with DO7 anti‐p53 (Sigma, Zwijndrecht, the Netherlands), OP64 anti‐CDKN1A (Calbiochem, part of Merck, Amsterdam, the Netherlands), 1501R anti‐actin (Millipore), and secondary polyclonal HRP goat anti‐mouse (Dako, Amstelveen, the Netherlands) antibodies. Membranes were incubated with ECL or ECL‐plus reagent (Amersham, Eindhoven, the Netherlands), and proteins were visualized using Hyperfilm ECL (Amersham). Films were digitalized in JPEG format and processed in paint.net. Band intensity was quantified using imagej (Abramoff *et al*., 2004).

### 2.7 Cell cycle analysis

A549, A549/PG13Luc, or IMR‐90 cells were harvested three or four days after siRNA transfection in 96‐well plates or 10‐cm dishes. Cells from 30 wells per condition were pooled, fixed in 70% ethanol, treated with RNase A (Sigma), and stained with propidium iodide (PI). Cells from 10‐cm dishes were harvested, fixed in 70% ethanol, and sequentially incubated with primary antibody against human phospho‐histone H3 (Ser10) (Millipore), secondary mouse anti‐rabbit Alexa 488 antibody, and PI/RNase staining buffer (BD Biosciences, Breda, the Netherlands). Alternatively, 1.5 h EdU incorporation was analyzed using the Click‐iT EdU Alexa Fluor 488 Imaging kit (Thermo Fisher Scientific). Data were acquired using a FACSCalibur (Becton Dickinson, Erembodegem, Belgium) and analyzed using modfit 4.05, applying the sync wizard (Verity Software house, Topsham, ME, USA), or with cellquest pro (Becton Dickinson).

### 2.8 Analysis of alternative splicing of *TP53*,* MDM2*, and *MDM4* genes

Cells were harvested 48 h after transfection with siRNAs targeting *SF3A3* (D‐019808‐03), *SNRPD3* (D‐019085‐04), *SF3B1* (D‐02061‐07), or *SF3B6* (D‐020260‐17) or nontargeting siRNA control NT#1. RNA was isolated using the miRNeasy mini kit (Qiagen, #217004) with an extra on‐column DNase digestion step (Qiagen, #79254) and an initial lysis step using TRIzol reagent (Thermo Fisher Scientific, #15596026). cDNA was prepared with M‐MLV Reverse Transcriptase (Solis BioDyne, Huissen, the Netherlands; #06‐21‐200000), random primers, dNTPs, and RNase out (all from Invitrogen). Real‐time PCR was performed on a Roche LightCycler 480 using the HOT FIREPol^®^ EvaGreen^®^ qPCR Mix Plus (no ROX) (Solis BioDyne, #08‐25‐00001). Primers were designed using primer‐blast 3.0 and purchased as custom oligonucleotides from Invitrogen. Primers specific for p53, MDM2, and MDM4 splice variants were designed to cover the exon junctions specific to the variants as shown in Table S2. Each qRT‐PCR experiment was performed in triplicate. Absolute expression levels were determined on the basis of the threshold cycle normalized to β‐actin (2^−ΔCt^). Relative expression levels were calculated relative to nontargeting siRNA controls using the ΔΔCt method.

### 2.9 Protein network and spliceosome pathway analysis

Primary hit selection was defined on the screen results for the positive p53 inhibitor control (*SYVN1*) and the positive p53 enhancer control (*TP53*). Those genes that had the empirical probability of being at least as strong as either control in all three screens were selected and uploaded to String‐db.org (v9.1). A high‐confidence (score 0.7) analysis was performed to find protein–protein interactions based on data from experiments, databases, and text mining.

The KEGG human spliceosome database (HSA03041) was downloaded from www.genome.jp/kegg/pathway.html, and average robust *Z*‐scores derived from the primary screens were matched with KEGG protein IDs.

### 2.10 Statistical analysis

High‐throughput p53 reporter screen data were processed in R using the CellHTS2 Bioconductor package (Boutros *et al*., 2006). Raw luminescence was log2‐transformed, normalized to mock per plate, and robust *Z*‐scores were calculated (Siebring‐van Olst *et al*., 2013). Fluorescence data from the cell viability screen were log2‐transformed, normalized to the negative control NTp2 per plate, and robust *Z*‐scores were calculated. In low‐throughput experiments, data were normalized to mock‐treated controls, and graphpad prism 6.0 (Graphpad Software, Inc., La Jolla, CA, USA) was used for statistical analysis. Differences in cell cycle distributions determined by flow cytometry were analyzed using ANOVA with Dunnett's correction for multiple testing. Differences in mRNA (variant) expression detected by quantitative real‐time PCR were tested by two‐sided Student's *t*‐test. In bar diagrams, data are presented as means + SD. Box plots are drawn according to Tukey. *P*‐values: *P < 0.05; **P < 0.01; ***P < 0.001; ****P < 0.0001.

## 3 Results

### 3.1 A genome‐wide siRNA screen identifies molecular targets for p53 activation

**Figure 1:**
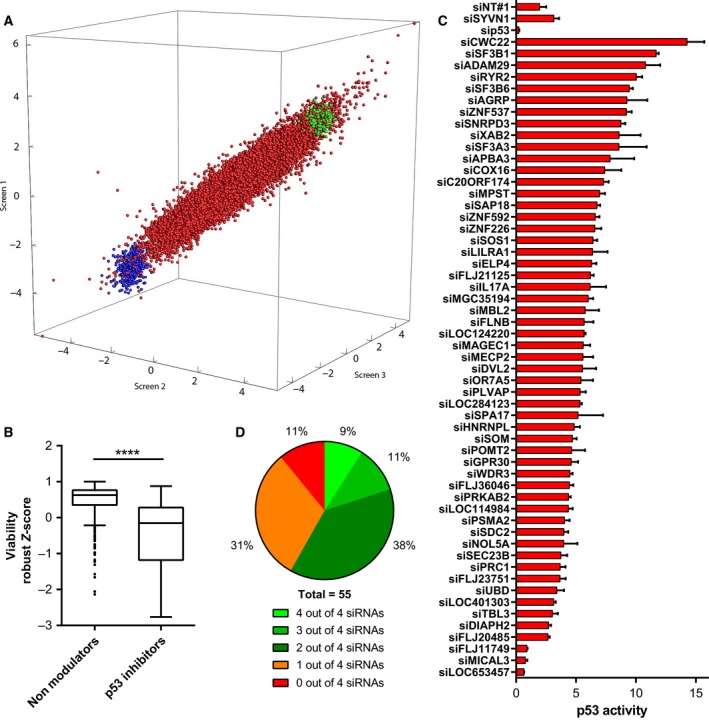
Whole human genome siRNA screens for p53 activity regulators. (A) 3D representation of the results of triplicate independent discovery screens on A549/PG13Luc cells. Each dot depicts the robust *Z*‐scores obtained for an individual sample. Red dots, library siRNA samples; green dots, si*SYVN1*; blue dots, si*TP53*. (B) Comparison of the viability of A549 cells transfected with siRNAs silencing 55 putative p53 pathway inhibitors versus 299 nonmodulators of p53 activity (Mann–Whitney test). (C) Relative p53 activity in A549/PG13Luc cells transfected with siRNA pools targeting the 55 strongest putative p53 pathway inhibitors identified in the genome‐wide screens. Data derived from two to four independent confirmation screens. (D) Four distinct siRNAs targeting the 55 strongest putative inhibitors were tested individually. Pie diagram depicts the number of siRNAs per target that consistently induced p53 activity at least twofold in two to four independent experiments. ****P < 0.0001

To identify molecular determinants of tumor suppressor p53 activity, we performed three independent genome‐wide siRNA screens on p53 wild‐type A549 NSCLC cells stable expressing a p53‐reporter construct (Siebring‐van Olst *et al*., 2013). Table S3 lists the normalized and scored data from the screens. Figure 1A shows the robust *Z*‐scores of the three screens, including positive control siRNAs targeting *TP53* and synoviolin (*SYVN1*), an ER‐resident E3 ubiquitin ligase that sequesters p53 in the cytoplasm and targets p53 for degradation (Yamasaki *et al*., 2007). We identified *SYVN1* in a pilot screen as a functional inhibitor of p53 activity in A549 cells (data not shown). As expected, silencing *TP53* decreased p53 activity (approximately fourfold; mean robust *Z*‐score −2.78) and silencing *SYVN1* increased p53 activity (approximately fourfold; mean robust *Z*‐score 2.62). Ninety genes that exhibited a mean *Z*‐score at least as low as that of *TP53* were considered putative p53 pathway enhancers; 592 genes with *Z*‐scores at least as high as that of *SYVN1* were considered putative p53 pathway inhibitors. The primary hit selection did not include paradigm p53 inhibitors MDM2 and MDM4. Silencing these genes did increase p53 activity (mean luminescence inductions 2.5‐ and 3.2‐fold, respectively), but with a mean robust *Z*‐score below 2.

The putative p53 pathway enhancers and inhibitors were subjected to STRING analysis for known and predicted protein–protein interactions. Analysis of 599 annotated genes eligible for network analysis revealed an interaction network consisting of 140 proteins (Fig. S1). In addition to positive control SYVN1, 16 other proteins were marked as directly interacting with p53. These included, for example, putative p53 pathway inhibitors SFN, STAT1, NCL, AKT1, PIAS4, and XPO1 and putative enhancers RPL5 and RPL11. While the identification of SFN and STAT1 as p53 pathway inhibitors was unexpected, as *SFN*‐encoded 14‐3‐3 sigma protein was reported to stabilize p53 enhancing its transcriptional activity (Yang *et al*., 2003) and STAT1 is known to act as a coactivator of p53 (Townsend *et al*., 2004), the identification of the other genes is in line with previous observations. NCL inhibits p53 mRNA translation (Takagi *et al*., 2005), AKT1 enhances p53 degradation (Ogawara *et al*., 2002), PIAS4 inhibits p53‐dependent CDKN1A and Bax transcription (Nelson *et al*., 2001), XPO1 mediates the nuclear export of p53 (Lain *et al*., 1999), and ribosomal protein (RP) L5 and RPL11 are part of a complex of RPs that inhibit p53 degradation (Dai and Lu, 2004). In the network, NCL, RPL5, and RPL11 connect to small clusters of identified proteins involved in ribosome biogenesis, including core components of RNA polymerase I and ribonucleoproteins required for ribosomal RNA processing and ribosome assembly. The largest cluster identified in our screen comprised many components of the mRNA splicing machinery, including, in particular, splice factor (SF)3 proteins and small nuclear ribonucleoprotein (SNRP) family members.

### 3.2 Hit selection and stratification of high‐confidence p53 pathway inhibitors

Hit selection for confirmation experiments was performed on the basis of robust *Z*‐scores (see Fig. 1A). A threshold *Z* > 3 in all three screens selected 55 siRNAs designed to silence annotated genes that increased p53 activity. We also identified eight siRNAs that decreased luciferase activity with a selection threshold *Z* < −3. Because the screens did not discriminate between decreased luminescence as a result of decreased p53 activity and decreased luminescence caused by loss of cell viability, we next stratified these siRNAs in a cell viability screen on A549 cells. Their effect on cell viability was tested against 299 control siRNAs that yielded a mean robust *Z*‐score around 0 in the p53 activity screen. Figure S2 shows that siRNAs against *OTUD7B* and *RTKN* decreased luminescence, but not cell viability. This verified that silencing *OTUD7B* and *RTKN* decreased p53 activity, suggesting that these genes encode p53 pathway enhancers.

As resurrecting wild‐type p53 in cancer cells could be of therapeutic benefit, we investigated whether silencing putative p53 pathway inhibitors affected A549 cell viability. The 55 siRNAs that increased p53 activity and the 299 siRNAs that exhibited robust *Z*‐scores around 0 were subjected to a cell viability screen (Fig. 1B). The siRNAs silencing putative p53 pathway inhibitors were enriched for siRNAs that decreased cell viability (*P* < 0.0001). Hence, induction of p53 activity in A549 cells by silencing putative p53 pathway inhibitors was associated with loss of cell viability.

Focusing on activation of p53 by inhibitor silencing, the 55 selected primary screen hits were stratified by performing confirmation screens with four distinct siRNAs targeting different sequences on the mRNA of these genes, as well as with pools of these siRNAs. Silencing 52 genes with siRNA pools resulted in an at least twofold p53 activity induction (Fig. 1C). For 32 genes, this could be reproduced with at least two individual siRNAs in all replicate screens performed (Fig. 1D and Table S4). On the basis of these observations, these 32 genes were considered to encode candidate inhibitors of p53 activity (Table 1).

**Table 1:** Molecular targets for p53 activation identified by siRNA library screening on A549 NSCLC cells

Gene symbol	Entrez gene ID	Average robust *Z*‐score	Fold induction p53 activity	# siRNAs
CWC22	57703	5.51	14.3	4
IL17A	3605	4.63	6.2	2
ADAM29	11086	4.48	10.8	2
XAB2	56949	4.37	8.6	4
SF3B6	51639	4.35	9.4	2
TSHZ3	57616	4.08	9.2	3
SF3A3	10946	4.00	8.6	3
SF3B1	23451	3.99	11.7	4
APBA3	9546	3.96	7.8	2
RYR2	6262	3.96	10.0	2
DVL2	1856	3.92	5.5	2
LILRA1	11024	3.90	6.4	2
SNRPD3	6634	3.85	8.7	3
NOP56	10528	3.78	4.0	3
OR7A5	26659	3.75	5.4	2
SAP18	10284	3.75	6.8	4
ZNF592	9640	3.66	6.6	3
COX16	51241	3.65	7.4	2
C1orf158	93190	3.63	6.0	2
SPA17	53340	3.63	5.2	2
ZNF226	7769	3.63	6.6	2
FLNB	2317	3.57	5.7	2
PLVAP	83483	3.56	5.3	2
CCDC116	164592	3.48	4.4	2
FLYWCH2	114984	3.42	4.4	2
GRHL3	57822	3.37	4.7	2
SOS1	6654	3.35	6.4	2
PSMA2	5683	3.31	4.0	2
FAM27L	284123	3.24	5.3	2
WDR3	10885	3.23	4.5	4
HNRNPL	3191	3.21	4.9	2
PUS7	54517	3.21	2.7	3

Average robust *Z*‐scores are derived from the primary screen; fold induction is derived from the secondary validation screen with pooled siRNAs. # siRNAs denotes the number of siRNAs out of four tested per candidate consistently inducing at least a twofold increase in p53 activity.

### 3.3 Validation of p53 pathway inhibitors

**Figure 2:**
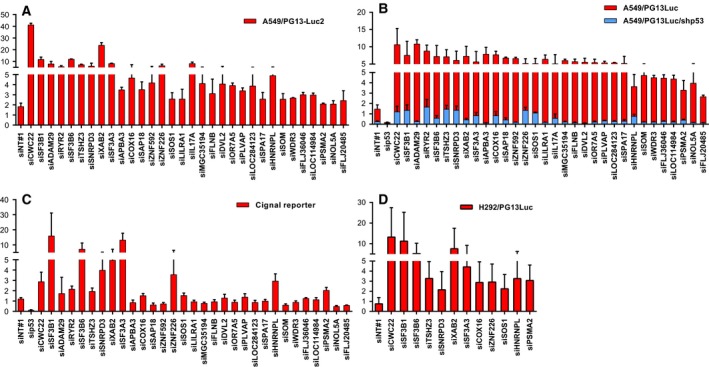
Validation of p53 pathway inhibitors on different NSCLC reporter cell lines. (A) Validation in independent A549 reporter cell clone A549/PG13Luc2. Data are derived from two independent experiments. (B) Validation of p53‐dependent luciferase induction. Comparison of p53 activity upon transfection of siRNAs silencing putative p53 pathway inhibitors into A549/PG13Luc cells and A549/PG13Luc/shp53 cells expressing a short hairpin targeting *TP53*. Data are derived from three independent experiments. (C) Validation using an independent p53 activity reporter system (Cignal assay). Data are derived from two independent experiments. (D) Validation in a second p53 wild‐type NSCLC cell line, NCI‐H292, carrying the PG13Luc reporter construct. Data are derived from three independent experiments. All candidate genes are presented in the same order as in Fig. 1C.

To independently validate the 32 candidate inhibitors of p53 activity, additional screens were performed on other NSCLC reporter cell lines. First, a distinct reporter cell clone was used (A549/PG13Luc2). Silencing of all candidate p53 pathway inhibitors induced luciferase expression also in this cell line (Fig. 2A). With a few exceptions, magnitudes of p53 induction followed a similar trend in the two A549/PG13Luc reporter cell clones. This made it unlikely that reporter gene expression was modulated via alternative transcription regulatory sequences near the insertion site of the reporter construct in the genome. Second, a derivative of A549/PG13Luc was made that expresses a short hairpin targeting *TP53*. Silencing of all candidate p53 pathway inhibitors in this cell line resulted in substantially reduced luminescence compared to A549/PG13Luc cells (Fig. 2B). This confirmed that activation of reporter gene expression was p53 dependent. Third, 31 siRNAs were tested on A549 cells using the Cignal p53 Reporter Assay. The reporter plasmid in this assay comprises a different p53 response element. IL17A was omitted from this analysis, because we could not detect any IL17A mRNA expression in A549 cells (Table S1). The Cignal assay proved less sensitive than the assay using PG13Luc reporter cells. Only 13 siRNA pools that were mostly among the strongest inducers of luminescence in cells carrying PG13Luc also induced luminescence in cells transfected with the Cignal p53 reporter construct (Fig. 2C). In our opinion, this does not disqualify the other 18 genes as inhibitors of p53 activity, but we decided to continue our studies only with the 13 genes that passed validation with both reporters. For these genes, knockdown was determined in siRNA‐transfected A549 cells using quantitative RT‐PCR and correlated with the luciferase expression (Table S1). Most genes were effectively silenced by multiple siRNAs. The exceptions were *RYR2* and *ZNF226*. *RYR2* was expressed at a very low level in A549 cells, and substantial knockdown was achieved with only one of the siRNAs used. *RYR2* was therefore excluded from further analysis. Silencing *ZNF226* was modest with variable induction of luciferase activity. Poor knockdown–phenotype correlation disqualified *ZNF226* as target for p53 pathway activation. For the other target genes, effective knockdown was generally associated with induction of luciferase expression. The exceptions were a single siRNA targeting *TSHZ3* and two siRNAs targeting *PSMA2*. *TSHZ3* was not considered disqualified, as knockdown at the mRNA level might not always translate in a similarly effective induction of the p53 pathway. In contrast, *PSMA2* was disqualified by the fact that while all four siRNAs achieved near complete knockdown, this was only associated with p53 activation in two cases. The 10 remaining candidates were tested in a distinct NSCLC cell line, NCI‐H292. Figure 2D shows that silencing of all 10 candidate p53 pathway inhibitor genes increased luminescence in H292/PG13Luc cells, validating their functional activity in multiple NSCLC cell lines.

**Figure 3:**
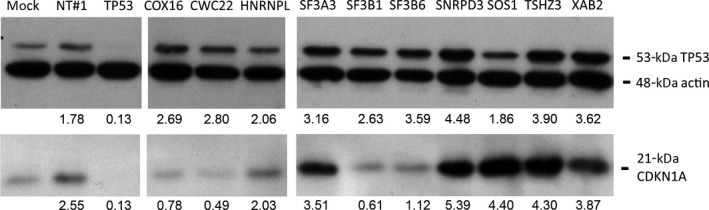
Western blot analysis of p53 and CDKN1A protein content in A549/PG13Luc cells transfected with siRNAs silencing p53 pathway inhibitor genes. Relative expression compared to the untreated control and normalized by the β‐actin loading control is depicted below each lane.

Further independent validation of the 10 candidates was determined by demonstrating endogenous p53 accumulation and CDKN1A (p21^WAF^) expression induction in A549/PG13Luc cells by western blot analysis (Fig. 3). A modest increase in p53 and CDKN1A protein content was observed upon transfection of nontargeting control siRNA. This possibly represents a nonspecific stress response induced by siRNA transfection or an unanticipated off‐target effect of the nontargeting control. In contrast, as expected, silencing *TP53* reduced p53 and CDKN1A protein levels. Compared to the nontargeting control, silencing candidate inhibitors of p53 activity increased p53 protein levels in A549 cells, except for *HNRNPL* and *SOS1*. For *SF3A3*,* SNRPD3*,* TSHZ3*, and *XAB2*, this was accompanied by an increase in CDKN1A expression. Interestingly, CDKN1A was not elevated upon silencing *COX16*,* CWC22*,* SF3B1*, and *SF3B6* despite clearly increased p53 levels. This was unexpected, in particular for *SF3B1* and *SF3B6*, as silencing these genes caused very high increases in p53 transcriptional activity in all independent reporter assays. In contrast, very strong CDKN1A expression was seen after silencing *SOS1*, while this did not elevate p53 protein expression.

### 3.4 Silencing p53 pathway inhibitors activates cell cycle checkpoints and promotes cell death

**Figure 4:**
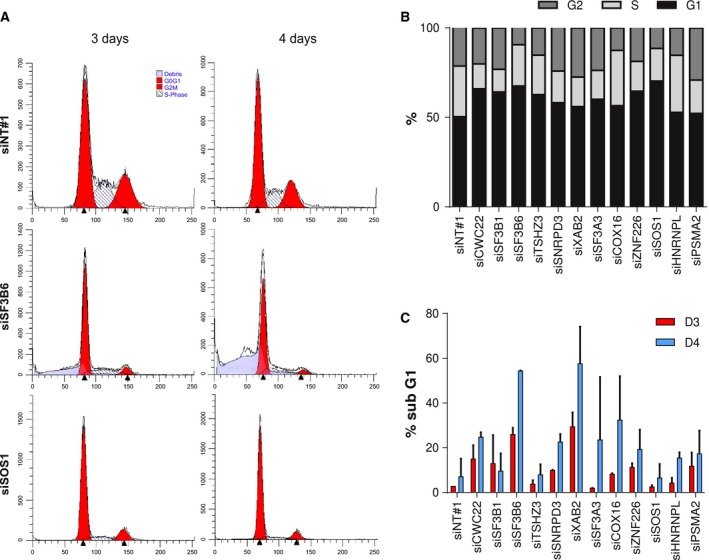
Effects of silencing candidate p53 pathway inhibitor genes in A549/PG13Luc NSCLC cells on the cell cycle. (A) Representative FACS DNA histograms (black lines) of A549 cells, three and four days after transfection with siRNAs targeting *SF3B6*,*SOS1*, or a nontargeting control siRNA. Events identified by ModFit analysis to represent cells in G0/G1 and G2/M phases are indicated in red; cells in S are indicated by the hatched area; cell debris is shown in purple. (B) Cell cycle phase distributions three days after transfecting siRNAs against all tested p53 pathway inhibitor genes. Data are the means of two independent experiments. (C) Percentage of cells in the sub‐G1 fraction, three and four days after siRNA transfection. Data are the means of two independent experiments.

**Figure 5:**
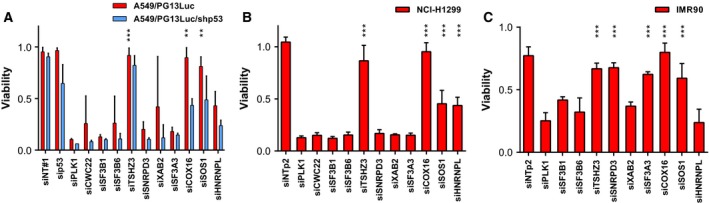
Effects of silencing candidate p53 pathway inhibitor genes on cell viability as determined by CellTiter‐Blue assay. Significance was tested against the siPLK1‐positive control by ANOVA with Dunnett's *post hoc* test. (A) Relative cell viability after siRNA transfection into A549/PG13Luc and A549/PG13Luc/shp53 cells. Data are derived from two to four independent experiments. Significance is given for A549/PG13Luc. (B) Relative cell viability after siRNA transfection into p53‐null NCI‐H1299 NSCLC cells. Data are derived from three independent experiments. (C) Relative cell viability after siRNA transfection into nonmalignant IMR‐90 lung fibroblast cells. Data are derived from three independent experiments. **P < 0.01; ***P < 0.001

To study the functional consequences of p53 activation by p53 pathway inhibitor silencing in A549 cells, we determined cell cycle distribution (Fig. 4). Silencing of most inhibitors of p53 activity, except *HNRNPL* and *COX16*, caused cell cycle arrest (Fig. 4A,B). Clear reductions were seen in the fraction of cells in S phase three days after siRNA transfection. In most cases, this was accompanied with an increase in G0/G1, indicating activation of the G1/S checkpoint. Furthermore, silencing of several genes induced A549 cell death, as evidenced by cells accumulating in sub‐G1 (Fig. 4C). This was corroborated by measuring reduced cell viability using the CellTiter‐Blue assay (Fig. 5A). The latter assay measures total metabolic capacity of the cultured cell population and does therefore not discriminate between cytostatic and cytotoxic effects. In these experiments, silencing *PLK1*, which is expected to cause cell death via induction of mitotic arrest (Liu and Erikson, 2003; Spankuch‐Schmitt *et al*., 2002), was included as positive cytotoxicity control. Silencing most candidate p53 pathway inhibitors induced A549 cell death also in this assay. To investigate whether the induction of cell death was p53 dependent, the CellTiter‐Blue assay was also performed on A549/PG13Luc/shp53 cells with reduced p53 activity (Fig. 5A) and on p53 null NCI‐H1299 NSCLC cells (Fig. 5B). Silencing p53 pathway inhibitor genes in these cell lines had similar effects on cell viability as observed on p53 wild‐type A549 cells. Hence, while silencing these genes activated p53, the associated induction of cell death did not require p53. To study the consequences of silencing the identified genes in healthy cells, IMR‐90 human lung fibroblasts were used (Fig. 5C). Endogenous expression of the identified genes as measured by qRT‐PCR was quite similar in A549 and IMR‐90 cells, with the exception of *TSHZ3* that was much higher expressed in IMR‐90 than in A549 cells (not shown). Nevertheless, IMR‐90 cells exhibited a different response to silencing several p53 pathway inhibitor genes than did A549 and NCI‐H1299 NSCLC cells. In particular, knockdown of the genes encoding splice factors SF3A3 and SNRPD3 was not toxic to IMR‐90 cells, whereas this effectively killed NSCLC cells. These genes could thus represent cancer‐selective therapeutic targets.

**Figure 6:**
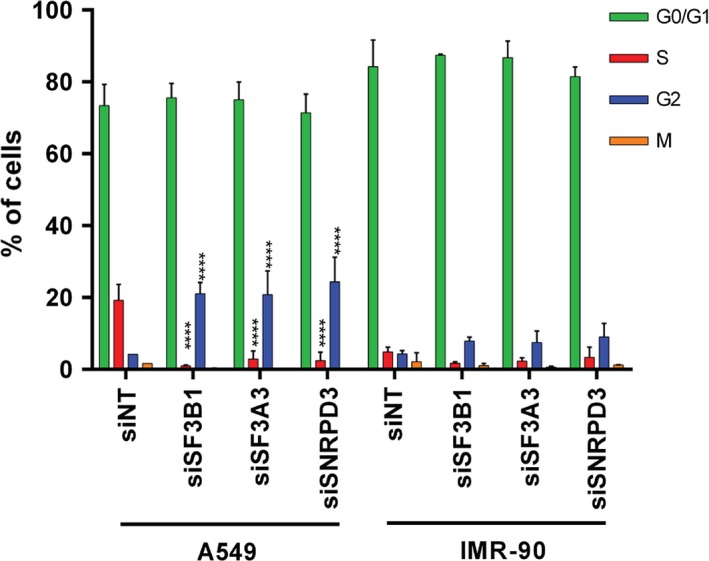
Effects of silencing splice factor genes on the cell cycle in NSCLC cells and lung fibroblasts. A549 and IMR‐90 cells were transfected with siNT, siSF3A3, siSF3B1, or siSNRPD3 and analyzed by flow cytometry three days later. Cells in different phases of the cell cycle were identified as shown in Fig. S3. Data are the means of two independent experiments. Error bars represent standard deviation. Significance of a change in the proportion of cells in each phase of the cell cycle following splice factor silencing was tested in comparison with cells transfected with nontargeting siRNA using ANOVA. ****P < 0.0001.

To obtain more insight into the differential effects of silencing *SF3A3* and *SNRPD3* on NSCLC cells versus fibroblasts, further cell cycle analyses were performed using EdU incorporation to detect active DNA synthesis in S‐phase cells; and immunostaining of histone H3 phosphorylation to dissect G2 and M phases of the cell cycle. Silencing *SF3B1*, toxic to both NSCLC cells and fibroblasts, was included as control. Figure S3 illustrates the gating procedure used in the flow cytometry experiments. As can be seen in Fig. 6, silencing splice factor genes in A549 cells reduced S phase and increased the fraction of cells in the G2 phase (*P* < 0.0001), indicating a strong inhibition of proliferation. This effect was also observed in IMR‐90 cells, but did not reach significance in these cells. For both cell lines, only very few cells were found in mitosis and this was not changed by splice factor silencing. The cytotoxicity of silencing splice factors thus appears associated with activation of cell cycle checkpoints, rather than defective mitosis.

### 3.5 Survey of the effects of silencing RNA splicing machinery components on p53 activity

**Figure 7:**
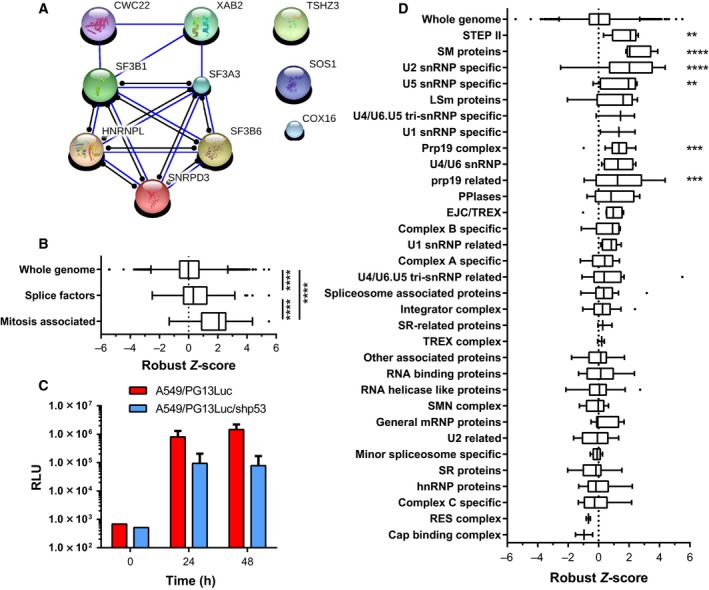
Analysis of p53 activity modulation by components of the RNA splicing machinery. (A) STRING network analysis of known and predicted protein–protein interactions between the 10 validated p53 pathway inhibitors. (B) Primary discovery screen scores for 342 canonical spliceosome pathway genes and a subset of splice factors involved in mitosis compared to the entire library gene set. (C) Induction of p53 activity in response to treatment with SF3b inhibitor pladienolide B. Luciferase expression in A549/PG13Luc and A549/PG13Luc/shp53 reporter cells at 0, 24, and 48 h after addition of 10 μm pladienolide B. Data are means + SD of two independent experiments performed in triplicate. (D) Primary discovery screen scores for 342 canonical spliceosome pathway genes, categorized into 32 functional groups, as defined in the KEGG pathway database. Data for each subgroup were tested against the whole genome using ANOVA with Dunnett's *post hoc* test. **P < 0.01; ***P < 0.01; ****P < 0.0001.

Our screen identified many putative p53 pathway inhibitors that are components of the mRNA splicing machinery. Seven of these were part of the top hits selected for further analysis. All seven passed every confirmation and validation experiment conducted, together finally constituting the majority of the validated hits (Fig. 7A). Thus, our screen for p53 pathway inhibitors appeared enriched for components of the mRNA splicing machinery. Splice factors are under consideration as targets for anticancer therapies. The observed induction of cell death and p53 transcriptional activity by splice factor silencing could thus be of relevance for the treatment of cancer. Therefore, we mined our primary discovery screen data for effects of components of the mRNA splicing machinery on p53 activity. Our siRNA library covered 342 of the 351 genes (i.e., 97%) defined as canonical spliceosome pathway members in the KEGG pathway database (Table S5). We tested the p53 activities measured upon silencing this gene set compared to the rest of the genome using the Mann–Whitney test and found that silencing spliceosome components generally increased p53 activity (Fig. 7B; *P* < 0.0001). Apart from their established function in pre‐mRNA splicing, several splice factors have been shown to be required for proper mitosis (English *et al*., 2012; Hofmann *et al*., 2010, 2013; Rines *et al*., 2008). We therefore separately tested this subset (Table S5). Silencing these 32 splice factors significantly activated p53 compared to the total set of canonical spliceosome pathway members (Fig. 7B; *P* < 0.0001), indicating that the subset involved in mitosis was further enriched for p53 pathway inhibitors. In fact, 10 splice factors of this subset were among the genes initially identified as putative p53 pathway inhibitors.

To further investigate the effect of spliceosome inhibition on p53 pathway activation, we treated A549/PG13Luc and A549/PG13Luc/shp53 reporter cells with the SF3b inhibitor pladienolide B (Kotake *et al*., 2007). As a missense mutation in SF3B1 could confer resistance against treatment with this compound, this splice factor is probably a direct target of pladienolide B (Yokoi *et al*., 2011). Within 24 h after pladienolide B addition, p53 activity rose almost 2000‐fold in A549/PG13Luc cells, further increasing to almost 3000‐fold after 48 h (Fig. 7C). In A549/PG13Luc/shp53 cells with reduced baseline p53 activity, pladienolide B treatment increased p53 activity to final levels approximately 19‐fold lower than observed in A549/PG13Luc cells. Thus, pharmacological inhibition of SF3B1 also potently induced p53 activity in A549 cells.

To obtain more insight into the elements of the spliceosome and the splicing regulatory proteins involved in p53 activation, the canonical spliceosome pathway members were categorized into functional groups and reanalyzed (Fig. 7D). Significant activation of wild‐type p53 was observed in particular upon silencing genes encoding Sm proteins (*P* < 0.0001), U2 snRNP‐specific proteins (*P* < 0.0001), Prp19 complex proteins (*P* = 0.0003), Prp19 complex‐related proteins (*P* = 0.0006), U5 snRNP‐specific proteins (*P* = 0.0037), and Step II factors (*P* = 0.0061).

### 3.6 Analysis of alternative splicing upon splice factor knockdown

**Figure 8:**
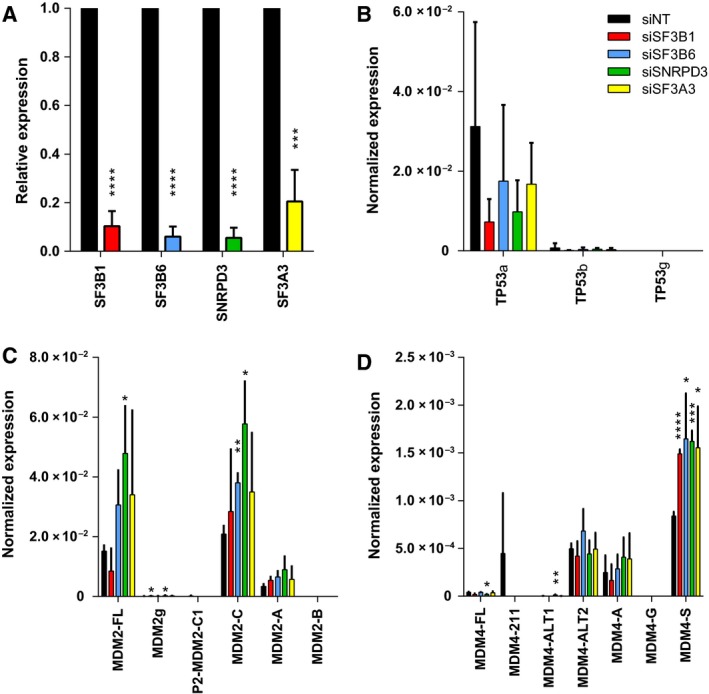
Analysis of alternative splicing of *TP53*,*MDM2*, and *MDM4* upon splice factor knockdown. A549 cells were transfected with siNT#1, siSF3B1, siSF3B6, siSNRPD3, or siSF3A3, and mRNA expression was quantified by qRT‐PCR. (A) Splice factor gene knockdown efficiency. For each splice factor gene, relative expression in specific knockdown cells versus siNT#1 transfected cells is shown. Panels B‐D show absolute expression normalized to β‐actin expression of *TP53* transcript variants (B), *MDM2* transcript variants (C), and *MDM4* transcript variants (D). Data are the means of three independent experiments. Error bars represent standard deviation. Significance of differential expression compared to nontargeting siRNA‐transfected cells was tested using Student's *t*‐test. *P < 0.05; **P < 0.01; ***P < 0.001; ****P < 0.0001.

To look into the mechanism through which silencing of splice factors might lead to activation of p53, alternative splicing of the mRNAs of p53 itself and of two paradigm p53 regulators, MDM2 and MDM4, was analyzed in A549 cells transfected with siRNAs targeting splice factor genes. Figure S4 shows the genome organization and known alternative splice variants for the three genes. Specific primers were designed to detect the α (full‐length), β (intron 9 retention), and γ (partial intron 9 retention) splice variants of p53 and all splice variants of MDM2 and MDM4 as described in the National Center for Biotechnology Information Gene database (see Table S2). Silencing of *SF3A3*,* SNRPD3*,* SF3B1*, or *SF3B6* (Fig. 8A) did not appear to have significant effect on expression of p53α, ‐β, or ‐γ mRNA variants (Fig. 8B). Silencing splice factors increased expression of *MDM2‐FL*,* MDM2g*, and *MDM2‐C* splice variants in some cases, but this did not always reach significance and did not change relative abundance of different splice variants (Fig. 8C). In contrast, significant and more consistent changes in splice variant expression were observed for *MDM4* (Fig. 8D). Silencing all four splice factors was associated with an approximately twofold increased expression of *MDM4‐S*, the most abundant *MDM4* variant detected in A549 cells (*P* < 0.05 to *P* < 0.0001). Silencing *SNRPD3* additionally changed expression of *MDM4‐FL* (twofold decrease, *P* < 0.05) and *MDM4‐ALT1* (40‐fold increase, *P* < 0.01) mRNA variants.

## 4 Discussion

In an effort to identify new therapeutic targets in NSCLC, we searched for therapeutic targets to reinstate functional p53 expression in NSCLC cells. To this end, we performed a genome‐wide siRNA screen on p53 wild‐type NSCLC cells with p53 transcriptional activity as readout. This approach does not discriminate between direct and indirect p53 activation. Our screen identified many genes previously unknown to influence p53 activity. Screens for p53 transcriptional activity covering a substantial part of the human genome were previously performed using cDNA and shRNA libraries in colon cancer cells and osteosarcoma cells, respectively (Huang *et al*., 2004; Llanos *et al*., 2006). In other screens, nonmalignant cells and bypass of p53‐dependent proliferation arrest as readout were used (Berns *et al*., 2004; Castro *et al*., 2008; Mullenders *et al*., 2009). Only one of our screen hits was reported in one of these earlier studies. This apparent discrepancy can be due to the cell type, readout method, or assay design used or could reflect differences in library performance. Nevertheless, our screen revealed a number of p53 pathway enhancers and appeared particularly potent in the identification of p53 pathway inhibitors.

We identified several known regulators of p53 activity, indicating the validity of our screen. Apart from these, we found many noncanonical p53 pathway inhibitor genes, some of which were independently validated in multiple NSCLC cell lines. Activation of p53 by silencing *SOS1* and the appearing underlying mechanism were particularly interesting observations. A549 cells carry mutant K‐Ras, which was reported to inhibit p53 via ATR and SNAIL (Lee *et al*., 2009), and silencing K‐Ras in A549 cells activates p53 (Tecleab *et al*., 2014). SOS1 functions to activate the Ras pathway in response to receptor tyrosine kinase activation by facilitating Ras–GTP association (Buday and Downward, 2008). Thus, silencing *SOS1* could activate p53 by releasing it from Ras‐mediated inhibition. However, constitutively active mutant K‐Ras has a very high affinity for GTP. In this context, modulation of Ras activity by SOS1 is likely less relevant. In fact, a small‐molecule inhibitor targeting SOS1 was shown to inhibit growth of cancer cells with wild‐type K‐Ras, but not cells with mutant K‐Ras, including A549 (Evelyn *et al*., 2014). Silencing *SOS1* was therefore not expected to activate p53 in A549 cells. In addition, SOS1 was unique among the p53 pathway inhibitors identified and characterized here in that its silencing had little effect on p53 level, but strongly induced CDKN1A expression and G1 cell cycle arrest. This suggests an effect of SOS1 on p53 activity other than via the alleged elimination of p53 protein through direct SNAIL binding in mutant K‐Ras cells (Lee *et al*., 2009). SOS1 could perhaps inhibit the binding affinity of wild‐type p53 for promoter sequences in its target genes, for example, by affecting p53 protein modification or cofactor recruitment.

Analysis of screen hits for predicted protein–protein interactions revealed two functional gene clusters, that is, ribosome biogenesis and mRNA splicing. The identification of genes involved in ribosome biogenesis is in line with a role for p53 in surveillance of coordinated ribosome assembly (Deisenroth and Zhang, 2010). Perturbation of ribosome biogenesis activates p53 via the RP‐MDM2‐p53 stress response pathway, where RPs including RPL5, RPL11, and RPL23 bind MDM2 blocking its E3 ubiquitin ligase function (Dai and Lu, 2004). Our screen hit list included mainly genes involved in ribosome biogenesis rather than structural components of the ribosome. This complements a previous finding that silencing of certain ribosome proteins selectively inhibits growth of p53 wild‐type cells (Krastev *et al*., 2011). It was suggested that interference with specific steps in the ribosome assembly process would propagate signaling through the p53 pathway. Our findings support this view. The identification of RNA polymerase I core components POLR1B and POLR1C as putative p53 pathway inhibitors was also expected. Pharmacological inhibition of RNA polymerase I with, for example, actinomycin D has been shown to increase p53 activity through the RP‐MDM2‐p53 stress response pathway (Ashcroft *et al*., 2000; Dai and Lu, 2004). Hence, silencing of *POLR1B* and *POLR1C* inhibiting *de novo* ribosomal RNA synthesis is likely to have a similar effect.

The most notable gene cluster identified in our screen included genes of the mRNA splicing machinery. All putative p53 pathway inhibitors from this cluster subjected to further analysis were validated as inhibitors of p53 transcriptional activity. Splicing of the vast majority of precursor mRNA (pre‐mRNA) into mature mRNA is catalyzed by the major spliceosome, a huge complex composed of five different small nuclear ribonucleoprotein (snRNP) subunits comprising small nuclear RNA (snRNA) together with their associated proteins and many non‐snRNP protein cofactors (Will and Luhrmann, 2011). Before spliceosome assembly, snRNAs are exported to the cytoplasm where they are combined with a ring of seven Sm proteins, producing a stable core snRNP that is subsequently reimported into the nucleus. Here, snRNP‐specific proteins bind onto the core snRNP to form the snRNP spliceosome subunits, which assemble on the pre‐mRNA in a sequential manner. During the process, the spliceosome consists of a changing set of subunits and different proteins are recruited in different stages. Extensive conformational rearrangements activate the spliceosome to perform the actual RNA splicing reaction, which is regulated by spliceosome‐associated proteins (SAPs), including serine‐/arginine‐rich (SR) proteins and heterogeneous nuclear ribonucleoproteins (hnRNPs).

We found that silencing of a variety of components of multiple structural spliceosome subunits activated wild‐type p53. In this respect, the effect of silencing genes encoding Sm proteins SNRPD1, SNRPD2, and top validated hit SNRPD3, or U2 snRNP‐specific factors, including several SF3a and SF3b proteins such as top validated hits SF3A3, SF3B1, and SF3B6, was particularly significant. Very strong p53 activation was also achieved by treating cells with the SF3B1 inhibitor pladienolide B. The Sm proteins are part of all core snRNPs and the U2 snRNP‐specific factors join the prespliceosome early in its assembly on the pre‐mRNA where they recognize the branch point sequence. They remain integral parts of the spliceosome throughout all steps of the splicing process. Thus, interfering with the early steps of spliceosome assembly was highly effective in triggering a p53 response. Nevertheless, p53 pathway inhibitors were also found among splice factors recruited to the spliceosome in later steps of its assembly. This included, for example, several U5 snRNP‐specific factors and Prp19/CDC5 complex proteins and related factors, including top validated hit XAB2. These proteins are thought to be involved in spliceosome remodeling during catalytic activation. Furthermore, several protein factors that are only present in the activated spliceosome, such as top validated hit CWC22, or even only during the second catalytic step completing the splicing process, were also found to inhibit p53 activity. Hence, prominent activation of wild‐type p53 was observed upon silencing of crucial components of early as well as late spliceosome assembly and activation. Interestingly, our hit list was enriched for splice factors previously reported to be essential for proper cell division. In particular, SNRPD3, CDC5L, EFTUD2, CDC40, XAB2, and CWC22 shown here to inhibit p53 activity were previously found to be required for mitosis (English *et al*., 2012; Hofmann *et al*., 2010, 2013; Rines *et al*., 2008). In addition, depletion of splice factors including SNRPD3, SF3B1, and XAB2 in cancer cells was found to cause defects in sister chromatid cohesion via aberrant splicing of soronin pre‐mRNA (Sundaramoorthy *et al*., 2014). It could be hypothesized, therefore, that the p53‐independent lethal effects that we observed upon silencing of a subset of spliceosome genes are explained by induction of mitotic catastrophe. However, in our experiments, silencing splice factor genes did not change the fraction of cells in mitosis. Instead, splice factor silencing consistently reduced the S‐phase fraction and increased G1 or G2 populations a few days after siRNA transfection, suggesting that activation of cell cycle checkpoints preceded the induction of cell death. This would be consistent with the notion that depletion of splice factors causes defects in multiple stages of the cell cycle, with more effective depletion causing disturbances in earlier cell cycle transitions (Karamysheva *et al*., 2015). In addition, silencing *SNRPD3* or *SF3A3* was in contrast to silencing *PLK1*, which is known to induce mitotic catastrophe, not toxic to nonmalignant fibroblasts. This suggests that the cancer‐selective killing effect of silencing these targets occurred via a process different from mitotic catastrophe.

Previously, Allende‐Vega *et al*. silenced the genes encoding six different splice factors, each representing a different spliceosome subunit or step in the spliceosome assembly, in cancer cells, and reported that this increased p53 level and transcriptional activity. Furthermore, pharmacological inhibition of SR protein phosphorylation had the same effect (Allende‐Vega *et al*., 2013). At first sight, their and our findings thus seem to suggest that p53 activation is a general consequence of interfering with the spliceosome. However, our results are partly conflicting with those of the previous study, leading us to a different conclusion. Of the six splice factors previously reported to modulate p53 activity, only SF3B1 and PRPF8 were identified as p53 pathway inhibitors in our screen. Furthermore, silencing of none of the SR proteins activated p53 in our study, contrasting the previous observation with an SR protein phosphorylation inhibitor. Hence, the spliceosome targets for p53 activation in NSCLC cells identified by us appeared largely distinct from those previously found in other cell types. In addition, Allende‐Vega *et al*. (2013) noted that inhibition of the spliceosome reduced *MDM4* mRNA expression and that silencing *SF3B1* altered the splicing of *MDM2* mRNA decreasing the expression of the full‐length protein. We did not observe these expression changes. Instead, we observed increased *MDM2* mRNA expression in several cases, but without a change in the balance between variants encoding proteins capable and incapable of p53 binding. Interestingly, we found that silencing all tested splice factors increased expression of the *MDM4‐S* exon 6 skipping variant. Although *MDM4‐S* encodes a protein with p53 inhibitory activity, high expression of *MDM4‐S* has been described to reduce expression of the full‐length protein causing an increase in p53 activity in cells with an intact p53 pathway (Bardot *et al*., 2015; Bezzi *et al*., 2013). Our observation that silencing splice factor genes causes an increase in *MDM4‐S* expression and activates p53 thus supports the hypothesis that alternative splicing of *MDM4* plays an important role in the activation of p53 in response to perturbation of the RNA splicing machinery (Bezzi *et al*., 2013).

The genome‐wide loss‐of‐function screening approach allowed us to obtain a comprehensive view on the effects of almost all proteins of the spliceosome on p53 activity in NSCLC cells. Although we identified many p53 pathway inhibitors among the proteins of the mRNA splicing machinery, silencing of the vast majority of splice factors did not influence p53 activity in NSCLC cells. Moreover, silencing different splice factors appeared to activate p53 in distinct manners. While p53 levels increased in almost all cases, this did not always result in increased CDKN1A levels. Together, our results suggest that at least in NSCLC cells the p53 pathway is activated at different levels by particular perturbations in the mRNA splicing machinery, rather than by general inhibition of the spliceosome. This argues against the possibility that induction of p53 is a general response to cellular stress induced by inhibition of proper mRNA splicing.

Silencing spliceosome components was associated with strong cytotoxicity to NSCLC cells. This cytotoxicity was p53 independent, showing that induction of cell death and activation of the p53 pathway were distinct phenomena. Nevertheless, this pointed at splice factors as putative therapeutic targets in NSCLC. In this respect, it is noteworthy that several natural and synthetic compounds targeting the spliceosome have been identified and shown to exhibit antitumor activity (Bonnal *et al*., 2012). In particular, compounds binding to SF3b subunit proteins, such as pladienolide B used herein, are gaining interest as potential new anticancer drugs. These compounds act by preventing binding of the U2 snRNP subunit to the pre‐mRNA, leading to inappropriate selection of the 3′ splice acceptor site and thus alternative splicing. The clear lethal effects of silencing several genes encoding SF3b proteins in NSCLC cells observed in our experiments suggest that these SF3b‐targeting compounds could indeed be useful for the treatment of NSCLC. However, although preclinical studies with currently available SF3b‐targeting compounds have shown that these are more toxic to cancer cells than to healthy cells, we found that silencing the expression of SF3B1 and SF3B6 proteins also caused considerable toxicity in nonmalignant IMR‐90 fibroblasts. Apparently, like cancer cells, IMR‐90 cells in culture could not cope with changes in mRNA splicing brought about by inhibition of SF3b function. This would suggest that the selective toxicity of SF3b‐targeting compounds is not caused by their molecular target specificity or by cancer cells being more vulnerable to inhibition of proper splicing. Instead, it could perhaps depend on the pharmacological properties of the drugs such as their cellular uptake or their target engagement under certain conditions. In contrast, we found that the silencing of at least two genes encoding proteins of other spliceosome subunits, that is, the Sm protein SNRPD3 and the SF3a subunit protein SF3A3, was much less toxic to fibroblasts than to NSCLC cells. This suggests that targeting other spliceosome proteins than SF3b proteins could perhaps be preferred. This warrants careful examination of the lethality of silencing individual spliceosome genes in relevant cell types, as this could contribute to the discovery of more selective anticancer agents targeting the spliceosome and ultimately to improved anticancer treatment.

## Author contributions

ES and VB designed and supervised the project. ESO, MB, and IM acquired the data. ESO, MB, RM, and VB analyzed and interpreted the data. ESO, MB, and VB wrote the draft manuscript. RM, IM, and ES reviewed and edited the manuscript.

## Supporting information


**Fig. S1.** STRING network of known and predicted protein–protein interactions between putative inhibitors and enhancers of p53 identified in the genome‐wide p53 transcriptional activity siRNA screen.
**Fig. S2.** Cell viability and p53 activity screen scores of putative enhancers of p53 activity in comparison with 299 irrelevant controls.
**Fig. S3.** Gating strategy for the flow cytometry cell cycle experiments.
**Fig. S4.** Exon–intron genome organization and known alternative RNA splice variants for human *TP53*,* MDM2* and *MDM4*.

**Table S1.** Knockdown‐phenotype analysis of individual siRNAs targeting 14 candidate p53 pathway inhibitors in A549/PG13Luc cells.

**Table S2.** Sequences and exon annealing positions of primers used in qRT‐PCR analysis of *TP53*,* MDM2* and *MDM4* splice variants.

**Table S3.** Primary p53 reporter screen results. The table lists the normalized luminescence values and the calculated robust *Z*‐scores of the three screens done.

**Table S4.** Results of deconvolution confirmation screens. The table lists the fold induction in luciferase expression measured in A549/PG13Luc cells upon transfection with four distinct siRNAs for each candidate target gene (2‐4 independent experiments per gene).

**Table S5.** p53 activity screen results (average robust *Z*‐scores) for canonical spliceosome pathway member proteins, with subgroup designation as defined in the KEGG pathway database, and splice factors with a previously reported role in mitosis.
