# Supplementary material for: A genome‐wide siRNA screen for regulators of tumor suppressor p53 activity in human non‐small cell lung cancer cells identifies components of the RNA splicing machinery as targets for anticancer treatment
Source: Mol Oncol. 2017 Apr 11;11(5):534–51. doi: 10.1002/1878-0261.12052 (PMC5527466; doi:10.1002/1878-0261.12052)
Supplement: Supplementary file 1 — Fig. S1. STRING network of known and predicted protein–protein interactions between putative inhibitors and enhancers of p53 identified in the genome‐wide p53 transcriptional activity siRNA screen. Fig. S2. Cell viability and p53 activity screen scores of putative enhancers of p53 activity in comparison with 299 irrelevant controls. Fig. S3. Gating strategy for the flow cytometry cell cycle experiments. Fig. S4. Exon–intron genome organization and known alternative RNA splice variants for human TP53, MDM2 and MDM4. [file MOL2-11-534-s001.pdf]

Supplementary Information

Supplementary Tables

Supplementary Table 1: Knockdown-phenotype analysis. The table lists the siRNAs transfected into A549/PG13Luc cells, the resulting inductions of luciferase expression measured and the gene knockdown efficiencies determined by qRT-PCR analysis using the primers shown.

Supplementary Table 2: Sequences and exon annealing positions of primers used in qRT-PCR analysis of *TP53*, *MDM2* and *MDM4* splice variants.

Supplementary Table 3: Primary p53 reporter screen results. The table lists the normalized luminescence values and the calculated robust Z-scores of the three screens done.

Supplementary Table 4: Results of deconvolution confirmation screens. The table lists the fold induction in luciferase expression measured in A549/PG13Luc cells upon transfection with four distinct siRNAs for each candidate target gene (2-4 independent experiments per gene).

Supplementary Table 5: Spliceosome: List of canonical spliceosome pathway member proteins with subgroup designation as defined in the KEGG pathway database that are represented in the siRNA library and their p53 activity screen results (average robust Z scores). Mitosis associated: list of splice factors with a previously reported role in mitosis and their p53 activity screen results (average robust Z scores). Results depicted NA represent the 9 canonical spliceosome pathway members that are not covered by the siRNA library used.

Supplementary Figures

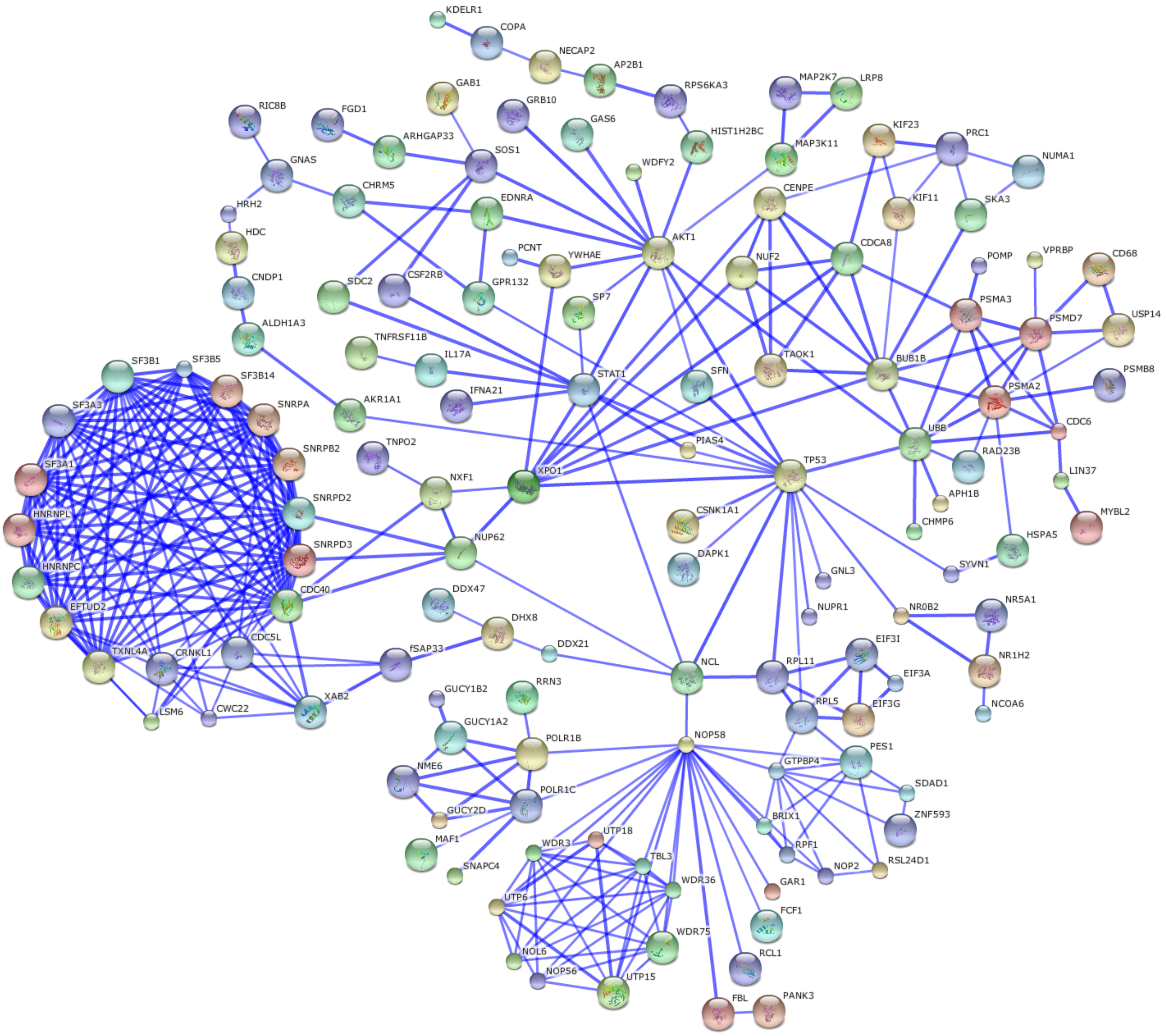

Supplementary Figure 1:

STRING network of known and predicted protein-protein interactions. Analysis on 592 putative inhibitors of p53 and 90 putative enhancers of p53 identified in the genome-wide p53 transcriptional activity siRNA screen in A549 cells. STRING analysis setting: high confidence (score 0.7).

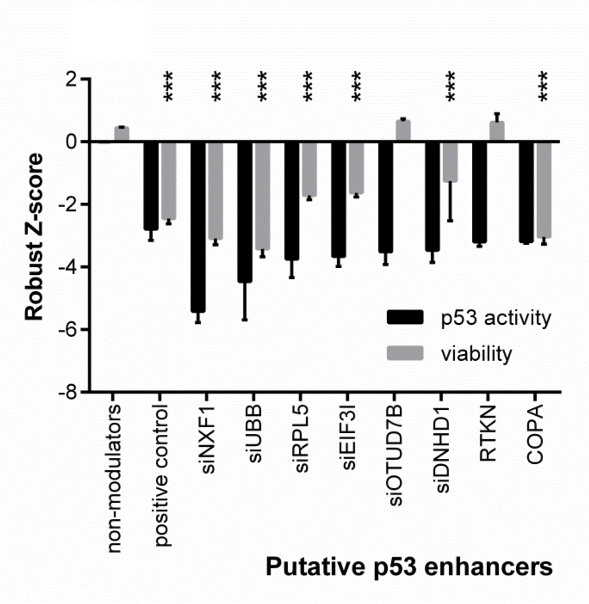

Supplementary Figure 2:

Cell viability and p53 activity screen scores of 8 putative enhancers of p53 activity in comparison to 299 irrelevant controls (i.e., genes with a robust Z-score 0 in the p53 activity screen). Black bars: results from the p53 luciferase reporter screen. The positive control in this screen depicted in this graph is silencing of *TP53*. Grey bars: results from the cell viability screen. The positive control in this screen is silencing of *PLK1*. Significance of change in cell viability was tested against the p53 non-modulators. ***, $p<0.001$.

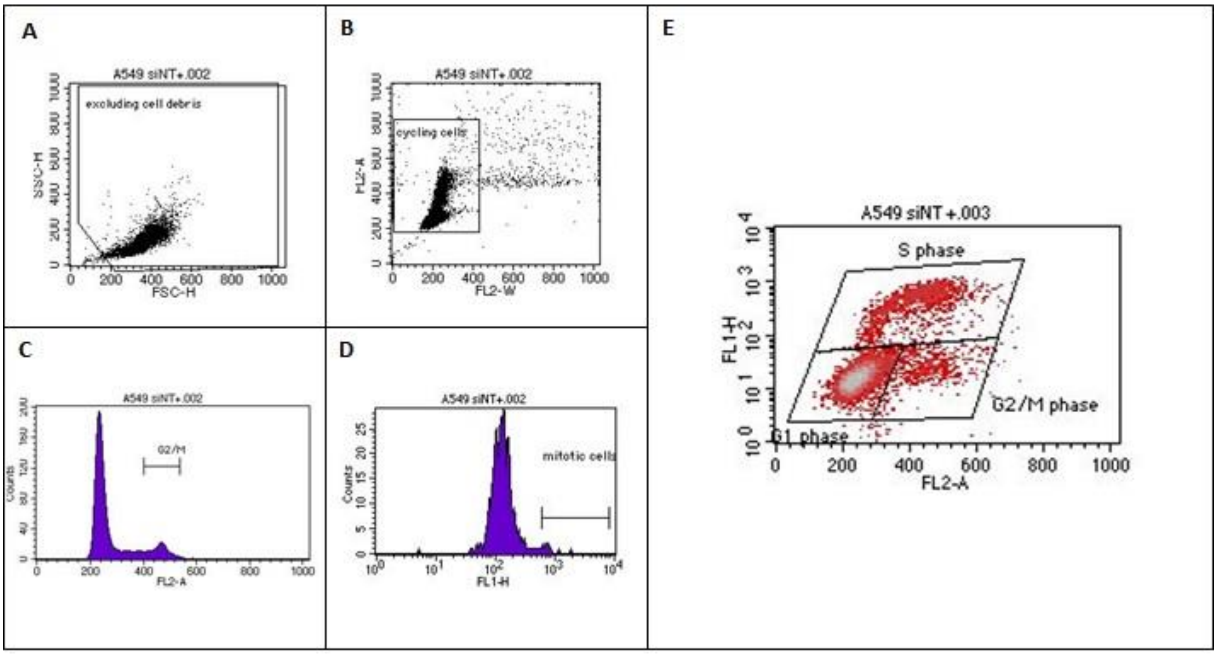

Supplementary Figure 3:

Gating strategy for the flow cytometry cell cycle experiments. Examplanary sample shown is A549 cells transfected with siNT. (A) exclusion of debris, based on forward-side scatter gating, (B) exclusion of cell douplets, based on PI area-width gating, (C) histogram of PI staining on cells gated according to panels A and B, with G2/M gating on the basis of DNA content, (D) phospho-histone H3 staining on G2/M phase cells gated in C, (E) cell cycle analysis by PI staining and EdU incorporation (cells gated according in A and B). The percentages cells in G0/G1 and S are derived from panel E; the percentages of cells in G2 and M are calculated from the G2/M population in panel E and the phospho-histon H3 staining in panel D.

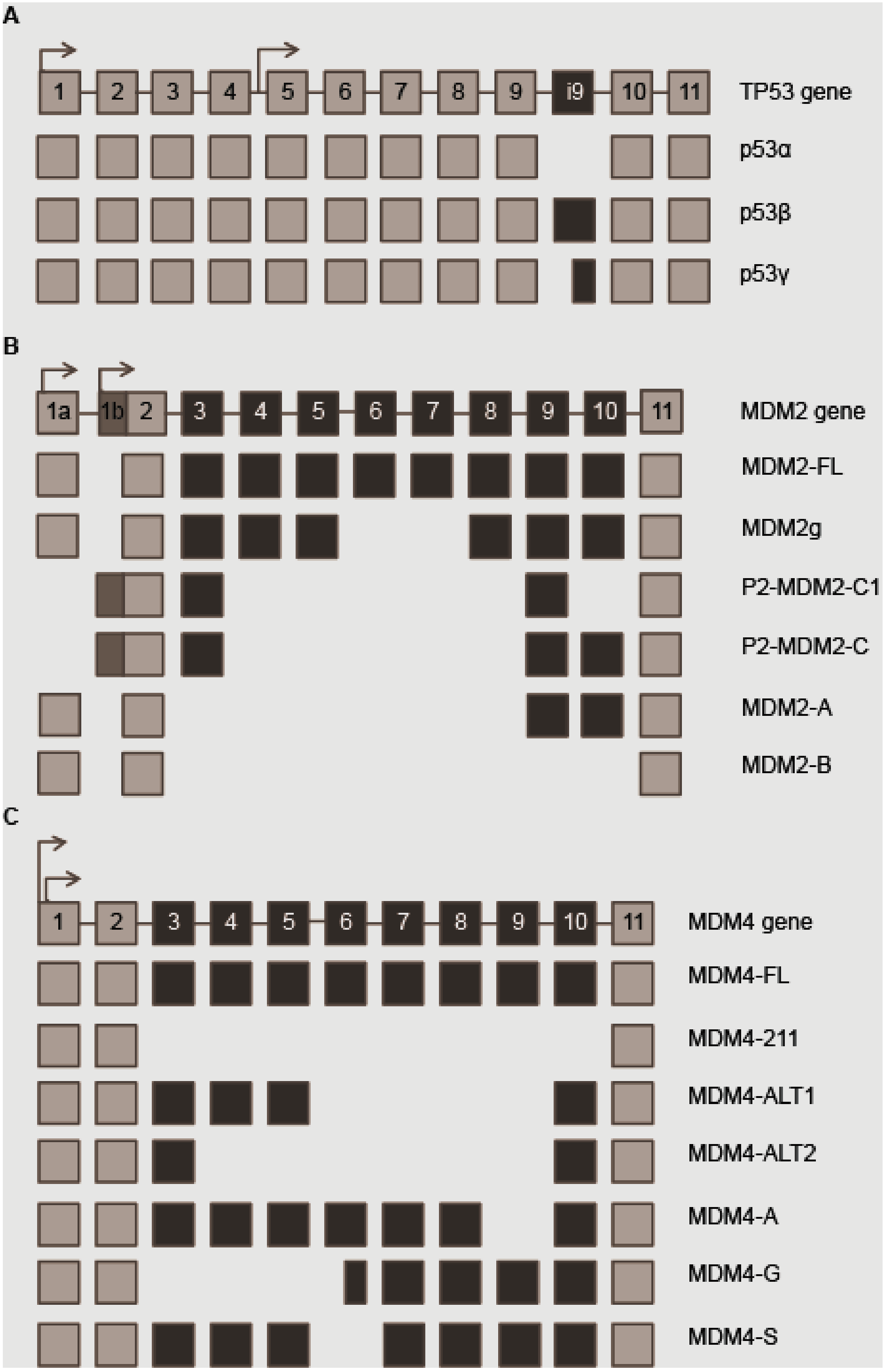

Supplementary Figure 4. Exon-intron genome organization and known alternative RNA splice variants for human *TP53* (A), *MDM2* (B) and *MDM4* (C). Intron (lines) and exon (boxes) lengths are not drawn to scale. Black boxes indicate exon inclusion/exclusion variants due to alternative splicing; dark gray boxes in *MDM2* indicate alternative exon 1 due to alternative transcription start at the second promoter in P2-MDM2-C(1) transcripts.
