# Supplementary material for: A genome‐wide siRNA screen for regulators of tumor suppressor p53 activity in human non‐small cell lung cancer cells identifies components of the RNA splicing machinery as targets for anticancer treatment
Source: Mol Oncol. 2017 Apr 11;11(5):534–51. doi: 10.1002/1878-0261.12052 (PMC5527466; doi:10.1002/1878-0261.12052)
Supplement: Supplementary file 3 — Table S2. Sequences and exon annealing positions of primers used in qRT‐PCR analysis of TP53, MDM2 and MDM4 splice variants. [file MOL2-11-534-s003.pdf]

| Gene_Splice_variant | Primer | Position_on_Exon_(junction) | Sequence (5' to 3') |
|---|---|---|---|
| p53α | forward | Exon 8 | GCTTTGAGGTGCGTGTTTGT |
| | reverse | Exon 9 – exon 10 | CGCCCACGGATCTGAAG |
| p53β | forward | Exon 8 | ATCTCCGCAAGAAAGGGGAG |
| | reverse | Exon 9 – intron 9 | TTTGAAAGCTGGTCTGGTCC |
| p53γ | forward | Exon 8 | ATCTCCGCAAGAAAGGGGAG |
| | reverse | Exon 9 – intron 9γ | TCGTAAGTCAAGTAGCATCTGAAGG |
| MDM2-FL | forward | Exon 8 – exon 9 | TCCGGATCTTGATGCTGGT |
| | reverse | Exon 10 | ATCACTCTCCCCTGCCTGAT |
| MDM2g | forward | Exon 5 - exon 8 | GTCAATCAGCAGGAAGAAAATTCAG |
| | reverse | Exon 8 – exon 9 | AAGATCCGGATTCGATGGCG |
| P2-MDM2-C1 | forward | Exon 3 | GGTGCTGTAACCACCTCACA |
| | reverse | Exon 9 - exon 11 | GTGCATTTCCAATAGTCCTCATCA |
| P2-MDM2-C | forward | Exon 3 – exon 9 | TATGAAAGAGGATCTTGATGCTGG |
| | reverse | Exon 11 | TCCCCTGCCTGATACACAGT |
| MDM2-A | forward | Exon 2 – exon 9 | AAGAGACCCTGGATCTTGATGCT |
| | reverse | Exon 11 | CACGAAGGGCCCAACATCT |
| MDM2-B | forward | Exon 2 – exon 11 | CAAGAGACCCTGGACTATTGGA |
| | reverse | Exon 11 | CCTCAACACATGACTCTCTGGA |
| MDM4-FL | forward | Exon 6 | AGATGCTGCTCAGACTCTCG |
| | reverse | Exon 8 – exon 9 | TGGCAGTACCCACATCCTGA |
| MDM4-211 | forward | Exon 2 – exon 11 | CTCCTGGACAAATCAATCAGGAAA |
| | reverse | Exon 11 | CCACTGAGTTGCAGGGATCA |
| MDM4-ALT1 | forward | Exon 3 | CAGGTGCGCAAGGTGAAATG |
| | reverse | Exon 5 – exon 10 | TCCCACTTCAATCACCTGTAGT |
| MDM4-ALT2 | forward | Exon 3 – exon 10 | ACTGTTAAAGAGGTGATTGAAGTGG |
| | reverse | Exon 11 | CCACTGAGTTGCAGGGATCA |
| MDM4-A | forward | Exon 7 | CACACTGCCTACCTCAGAGC |
| | reverse | Exon 8 – exon 10 | CCCACTTCAATCACCTGATTTGTC |
| MDM4-G | forward | Exon 2 – exon 6 | CCTGGACAAATCAATCAGGATCAC |
| | reverse | Exon 7 | AGGTAGGCAGTGTGGGGATA |
| MDM4-S | forward | Exon 3 | CAGCAGGTGCGCAAGGTGAA |
| | reverse | Exon 5 – exon 7 | GCACTTTGCTGTAGTAGCAGTG |
| β-actin | forward | Exon 3 | TTCCTATGTGGGCGACGAG |
| | reverse | Exon 3 | TCCTCGGGAGCCACACG |
